# Psychopathological Symptoms and Gaming Motives in Disordered Gaming—A Psychometric Comparison between the WHO and APA Diagnostic Frameworks

**DOI:** 10.3390/jcm8101691

**Published:** 2019-10-15

**Authors:** Christian Montag, Bruno Schivinski, Rayna Sariyska, Christopher Kannen, Zsolt Demetrovics, Halley M. Pontes

**Affiliations:** 1 Department of Molecular Psychology, Institute of Psychology and Education, Ulm University, 89081 Ulm, Germany; rayna.sariyska@uni-ulm.de; 2 School of Media and Communication, RMIT University, VIC 3000 Melbourne, Australia; bruno.schivinski@gmail.com; 3 The International Cyberpsychology and Addictions Research Laboratory (iCARL), University of Tasmania, TAS 7005 Launceston, Australia; 4 Independent Researcher: 50226 Frechen, Germany; info@ckannen.com; 5 Institute of Psychology, ELTE Eötvös Loránd University, 1064 Budapest, Hungary; 6 Division of Psychology, School of Medicine, University of Tasmania, TAS 7005 Launceston, Australia

**Keywords:** gaming disorder, internet gaming disorder, world health organization, American psychiatric association

## Abstract

Background: ‘Gaming Disorder’ (GD) has received increased medical attention and official recognition from both the American Psychiatric Association (APA) and the World Health Organization (WHO). Although these two medical organizations have independently developed promising clinical diagnostic frameworks to assess disordered gaming, little is known about how these frameworks compare at different psychometric levels in terms of producing consistent outcomes in the assessment of GD. Methods: A sample of 1429 German gamers (Mean_age_ = 29.74 years; SD = 12.37 years) completed an online survey including measures on different psychopathological symptoms (depression, loneliness and attention problems), gaming motives and disordered gaming according to the WHO and APA frameworks. Results: The findings suggest the existence of minor discrepancies in the estimation of prevalence rates of GD according among the two frameworks. Nevertheless, both diagnostic frameworks are fairly consistent in the psychometric prediction of GD in relation to gaming motives and psychopathological symptoms. The findings underscore the role of key gaming motives as risk factors and protective factors across both diagnostic frameworks. Finally, the study provides support for the WHO diagnostic framework for GD and its measurement with the German Gaming Disorder Test (GDT). The findings and their implications are further discussed in terms of clinical relevance.

## 1. Introduction

Research on the addictive effects of video games has recently witnessed key major developments at both theoretical and clinical levels that played a significant role in the recent official recognition of ‘Gaming Disorder’ (GD) as an official behavioral addiction by the World Health Organization (WHO) [1]. According to Pontes and Griffiths [2], the first major milestone for GD research took place in 2013 when the (fifth) edition of the Diagnostic and Statistical Manual of Mental Disorders (DSM-5) published by the American Psychiatric Association (APA) tentatively included ‘Internet Gaming Disorder’ (IGD) as a clinical phenomenon in need of further research [3]. Following this preliminarily recognition, the second milestone occurred when GD emerged in the beta draft of the 11th edition of the International Classification of Diseases (ICD-11) in 2016 [4]. Finally, the third key milestone took place in 25 May 2019 at the 72nd World Health Assembly which culminated in the historic and long-awaited decision by the WHO recognizing GD as a mental health disorder [2].

In terms of clinical presentation and diagnostic features, the APA [3] suggested that the clinical diagnosis of IGD comprises a behavioral pattern characterized by persistent and recurrent use of games, leading to significant impairment or distress over a period of 12 months as indicated by the endorsement of five (or more) of nine diagnostic criteria. The nine proposed criteria for IGD include: (1) preoccupation with games; (2) withdrawal symptoms when gaming is taken away; (3) tolerance, resulting in the need to spend increasing amounts of time engaged in games; (4) unsuccessful attempts to control participation in games; (5) loss of interest in previous hobbies and entertainment as a result of and with the exception of, games; (6) continued excessive use of games despite knowledge of psychosocial problems; (7) deceiving family members, therapists or others regarding the amount of gaming; (8) use of games to escape or relieve negative moods; and (9) jeopardizing or losing a significant relationship, job or education or career opportunity because of participation in games. At more severe levels, IGD may lead to academic failure, job loss or marriage failure as the problematic behavior tends to displace usual and expected social, work and/or educational, relationship and family activities. Interestingly, the WHO suggested a different conceptualization and diagnostic approach for GD by defining it as a problematic pattern of gaming behavior marked by (1) impaired control over the gaming activity; (2) increasing priority to the extent that gaming takes precedence over other interests and daily activities; and (3) continuation or escalation of the gaming activity despite the occurrence of negative consequences. Although GD involves recurrent gaming behavior which may be online or offline, its clinical symptoms must be assessed within a 12-month timeframe and be of sufficient severity to result in significant impairment across different life domains including personal, family, social, educational, occupational and/or other broad areas of functioning [1]. The WHO further specifies that differential diagnosis of GD can be achieved by screening for the following exclusion criteria: hazardous gaming, bipolar type I and type II disorders [4].

Recent research has suggested that the marked difference in the conceptualization and number of clinical criteria needed to be endorsed to diagnose IGD and GD may lead to potential biases in future research aiming at establishing prevalence rates, risk factors and clinical course of GD [5]. To the best of the authors’ knowledge, only one recent study [6] has examined potential differences in relation to the APA and WHO diagnostic framework. In their study, Jo and colleagues [6] compared the clinical features and gaming behaviors between gamers that were simultaneously diagnosed with the nine IGD criteria (as proposed by the DSM-5) and the GD criteria (as proposed by the ICD-11) so participants could be classed either as ‘normal’ (*n* = 115) or ‘DSM-5 + ICD-11’ (*n* = 12). The results of this study suggested that the clinically diagnosed sample presented significantly higher levels of depressive, oppositional defiant disorder and conduct disorder symptoms in comparison to participants in the ‘normal’ group. Although the prevalence rate of GD reported in this study was of 6.4%, these findings come with an important caveat as the differences in the experience of psychopathological symptoms and prevalence rates cannot be understood comparatively in relation to the APA and WHO diagnostic frameworks as the study used a combined clinical diagnosis of GD. Therefore, further research exploring the potential nuanced psychometric differences between the two diagnostic frameworks is warranted.

Notwithstanding this, current data suggests that GD affects a relatively small fraction of gamers, potentially ranging anywhere from 0.7% in Western countries [7] to 4.0% in Asian countries [8]. Even though the existing figures concerning prevalence rates of GD are limited due to methodological inconsistencies in the assessment of GD [9], researchers have suggested that such prevalence rates would rarely be above 5% in robust studies using large and representative data and appropriately validated psychometric tests to assess GD [10]. As recently outlined by Griffiths and Pontes [11], important gaps in research need to be addressed if this emerging field is to progress further, including examining potential biases in prevalence rates stemming from the adoption of a specific diagnostic framework in favor of another. Based on this, it has been reported [11] that current research on GD denotes a scarcity of systematic empirical data and robust comparative evidence in relation to both APA and WHO diagnostic frameworks in terms of (i) the key psychological motives for engaging with gaming in a dysfunctional pattern; (ii) the best practices for cross-cultural psychometric assessment; and (iii) the actual interplay between psychopathological symptoms and GD taking into account potential mediational effects of specific psychological motives for engaging with gaming. It is paramount to review and further investigate existing psychometric tools and diagnostic criteria commonly adopted in the assessment of GD as such practices can help generating consensus on the diagnosis of GD whilst identifying discrepancies in current GD frameworks as such potential discrepancies can effect prevalence rates estimates, diagnostic and treatment procedures for GD [12]. Such analyses are particularly relevant when conducted in a comparative way as opposed to individual analyses of specific diagnostic frameworks carried without context or reference to alternative diagnostic frameworks. Moreover, such comparative analyses can be made in relation to psychological motives and how they may precipitate disordered gaming symptoms.

### 1.1. Psychological Motives in Gaming-Behaviors

Empirical research examining the psychological motives for playing video games has been conducted for over two decades now. Indeed, this is an important aspect to be investigated at the psychological level as a comprehensive understanding about the nature of gaming activities (online and offline) and the specific factors leading gamers to engage with the activity can shed further light on the potential underlying mechanisms of healthy, excessive and disordered gaming.

Although the first studies on player motives focused on establishing general taxonomies of Multi-User Dungeon (MUD) players [13], subsequent research conducted by Yee [14] attempted to develop a broader framework to further the understanding about the role of gaming motives. More specifically, Yee [14] examined data from 3000 online gamers engaged in Massively-Multiplayer Online Role-Playing Games (MMORPGs) to establish through a factor analytic approach an empirically grounded model of player motives. Based on this research, a total of 10 specific gaming motives were found and clustered on three broad domains (i.e., achievement, social and immersion). More specifically, achievement described specific in-game motives featuring advancement, mechanics and competition motives while the social domain included general in-game socializing, relationship formation and teamwork motives. Finally, Yee’s model [14] described the immersion domain which relates to in-game discovery, role-playing, customization and escapism motives.

Despite these important early advancements in the psychological research of gaming motives, much was still left to be understood in terms of how gaming motives manifested throughout the wider spectrum of game genres as earlier research focused essentially on developing typologies of gamers and uncovering gaming motives for specific types of games (e.g., MUDs, MMORPGs). Therefore, further research by Demetrovics and colleagues [15] attempted to address this important shortcoming and capture the components of the motivational basis of games whilst developing the Motives for Online Gaming Questionnaire (MOGQ) to measure the key dimensions of gaming motives. After analyzing data from 3818 gamers, Demetrovics and colleagues [15] were able to identify seven primary motivational factors in gaming behavior applicable to all types of online games. Accordingly, these included social, escape, competition, coping, skill development, fantasy and recreation gaming motives. More specifically, social motives highlight players’ positive experiences in getting to know people, being with others and playing together with other gamers. Moreover, escape motives denote the desire to evade from reality and avoid problems in the real world. Competition motives include the desire to compete with and defeat other players in order to experience a sense of achievement. Coping motives reflect the players’ desire to use games to cope with distress and enhance their mood. Skill development motives characterize the players’ desire to engage in gaming to improve their skills (e.g., coordination, concentration or other abilities). Fantasy describes the motive of stepping out of one’s usual identity, trying new identities in a different fantasy world and trying new in-game experiences that cannot be done in real life. Finally, recreation motives relate to players’ need to achieve relaxation through gaming.

### 1.2. Gaming Motives and Psychopathological Symptoms among Gamers

Although several studies have focused on investigating the psychological motives underpinning healthy and excessive gaming for example, References [16,17,18], little research on specific gaming motives has been conducted to explore the potential role of gaming motives in predicting and mediating disordered gaming and key psychopathological outcomes. This is particularly concerning given that previous research has found that specific psychological motivational factors have an important role in the development and treatment of addictive disorders with and without the use of psychoactive substances [19,20,21,22,23].

Despite the fact that a few studies have been conducted to investigate the role of specific gaming motives in the development of GD, inconsistencies warranting further clarification still exist in the literature. For example, Ramos-Diaz and colleagues [24] recently reported that when gamers play for social motives, they present decreased risk for the development of GD. Moreover, motivations linked to escape and fantasy motives have been identified as the strongest predictors of GD in their sample [24]. Conversely, Dauriat and colleagues [25] found that social motives were linked to the experience of increased symptomatology of GD alongside, escape and achievement motives. Additionally, subsequent research has shown that social motives were associated with higher levels of GD in comparison to all other traditional gaming motives [26]. A further large-scale study conducted Király and colleagues [27] on a sample of 3186 online gamers found strong evidence suggesting that overall psychological distress has a significant direct and indirect effect on GD via escape and competition motives, further underscoring the fact that gaming motives may play a key role in the complex relationship between psychopathological distress and GD.

In terms of the specific relationship between gaming motives and GD, a relatively large body of studies have successfully demonstrated the role of the escape motive as the strongest predictor of GD in comparison to all other motives [26,28,29]. A recent longitudinal study [30] examining the link between gaming motives and GD according to distinct latent profiles of gamers (i.e., high-engagement, medium-engagement, low-engagement and healthy-engagement) reported that high-engagement gamers exhibited greater levels of advancement and escape motives and moderate levels of social motives in comparison to all other latent profiles found in the sample. By contrast, healthy-engagement gamers demonstrated the highest levels of well-being and social motives, moderate levels of advancement and low levels of escape motives. In comparison to healthy-engagement, high-engagement gamers exhibited significantly higher risk of experiencing psychological distress, further showing the potential for the development of psychological maladjustment [30]. In a similar vein, a nationally representative study of 1401 Koreans aged between 18 and 74 years old [29] reported that the escape motive was significantly associated to depression and greater severity of GD, showing that adults with depression may resort to gaming to escape from adverse emotions, further exacerbating excessive gaming behaviors that can develop into a full-blown addictive behavioral pattern in relation to gaming.

Another key aspect of gaming motives is related to its potential role in the treatment of GD. Although there is a paucity of empirical research examining the interplay between gaming motives and GD treatment, a recent study [31] focusing on establishing the links between gaming motives, related real life activities and treatment approaches for GD reported that socially motivated gamers tend to greatly enjoy social activities in real life, despite the fact that they may show decreased interest in creative activities. In a similar vein, immersion-oriented gamers display great interest in intellectual activities, with reduced interest in social activities and vacationing. These findings are important because they can inform decisions from practitioners when making targeted recommendations of replacement behaviors for disordered gamers based on their own unique motivational profile, which is key for effective diagnosis and treatment of GD [19,20,21,22,23].

### 1.3. The Current Study

Based on the literature reviewed, the aim of the present study was twofold. Given the obvious discrepancies in the two GD diagnostic frameworks put forth by the APA and the WHO (i.e., different focus on core diagnostic criteria and different number of criteria needed to be endorsed for diagnosis), the first aim of the present study was to provide a robust psychometric comparison of both diagnostic frameworks in terms of how GD can be explained by psychopathological symptoms through specific gaming motives and at the same time investigate potential effects in relation to GD prevalence rates stemming from the choice of the diagnostic framework. To the best of the authors’ knowledge, no previous study has compared the effectiveness of the two GD frameworks in relation to psychopathological symptoms, gaming motives and prevalence rates.

## 2. Methods

### 2.1. Data Cleaning and Management

The present study included a large sample of gamers (aged > 12 years) recruited via the German platform www.gaming-disorder.org. The project was initially registered at the Open Science Framework (OSF) platform and the dataset made freely available to ensure greater transparency and better reproducibility [32]. Participants aged between 12 to 18 years were required parental permission to partake in the present study. An initial assessment of the dataset led to the exclusion of 134 participants due to several reasons, including but not limited to: lack of parental consent and/or problematic responding patterns (e.g., endorsing sham items, providing unreasonable amounts of time spent gaming, and/or reporting not having played games in the last twelve months).

Although the present study did not provide any financial incentives to participants, those fully completing the survey received detailed graphical and text-based anonymized feedback containing data-driven insights into their own gaming patterns and related behaviors. All participants were assured of anonymity and confidentiality and the study was granted ethical approval by the research team’s University Ethics Committee (*N°: 2018/95*). Additionally, all procedures of this study were carried out in accordance with the ethical standards of the responsible committee on human experimentation and with the Helsinki Declaration of 1975, as revised in 2005. After the data cleaning procedures, the final sample consisted of 1429 eligible participants.

### 2.2. Measures

#### 2.2.1. Sociodemographic and Gaming-Related Variables

Data on the sample’s main sociodemographic characteristics and gaming-related behaviors were collected. These included gender, age, relationship status, employment status, time spent gaming (weekdays and weekends), preferred mode of play (online; offline; or both) and self-report assessment of significant problems due to gaming (yes/no).

#### 2.2.2. Disordered Gaming

To measure disordered gaming through the APA and WHO diagnostic frameworks, the Gaming Disorder Test (GDT) [5] and the Internet Gaming Disorder Scale–Short-Form (IGDS9-SF) [33] were employed (see Supplementary File for further information on the psychometric properties of the German GDT and IGDS9-SF). The GDT consists of four items answered on a five-point Likert scale ranging from 1 (‘never’) to 5 (“very often’) developed to assess symptoms of GD in the past 12 months according to the WHO diagnostic framework. Total scores are obtained by totaling up all four items (range = 4 to 20 points), with higher scores indicating greater levels of GD. A participant may be classed as disordered gamer by answering all four items either with ‘often’ (4) or ‘very often’ (5). Previous research reported excellent psychometric properties for the GDT [5]. The APA diagnostic framework for disordered gaming was assessed using the IGDS9-SF, which contains nine items used to assess the severity of disordered gaming and its detrimental effects of gaming activities occurring over a 12-month period. The nine items of the IGDS9-SF are answered using a five-point Likert scale ranging from 1 (‘never’) to 5 (‘very often’). Total scores for the IGDS9-SF can be obtained by summing the participants’ answers to all nine items (range = 9 to 45 points), with higher scores being indicative of higher degrees of disordered gaming. The IGDS9-SF has been investigated across multiple cross-cultural studies and has been reported to be a valid and reliable tool across different countries and samples [34,35].

The choice to assess disordered gaming symptoms with the IGDS9-SF was informed by the large body of existing evidence supporting its use across different cultural contexts alongside its ability to measure the nine IGD criteria effectively. More specifically, the IGDS9-SF was the first brief psychometric tool published to assess IGD symptoms based on the DSM-5 criteria and it has received the most cross-cultural support in comparison to alternative IGD-based tools as it has been psychometrically validated in a number of languages, such as Albanian [36], Chinese [34], English [33], Slovenian [37], Italian [38], Persian [39], Polish [40], Portuguese (European) [41] and Turkish [42]. In relation to its diagnostic performance, the IGDS9-SF has been investigated in both clinical and non-clinical samples. A study by Monacis and colleagues [38] in a sample of 757 Italian gamers found through a Receiver Operating Characteristic (ROC) curve analysis that the optimal cut-off point for the IGDS9-SF was 21, which resulted in a Positive Predictive Value of 96.85%, Negative Predictive Value of 55.31%, Accuracy of 86.02%, Sensitivity of 86.10% and Specificity of 86.0%. In clinical samples, a recent study by Severo and colleagues [43] on a subsample of clinically diagnosed disordered gamers (*n* = 104) found that the optimal cut-off point for the IGDS9-SF was > 21, which resulted in a Positive Predictive Value of 27.78%, Negative Predictive Value of 100%, Accuracy of 85.50%, Sensitivity of 100% and Specificity of 86.87%. Furthermore, the GDT has been adopted for the present study as there is no other existing measure for GD based on the recent framework developed by the WHO.

##### Gaming Motives

The German version of the MOGQ [15] was adopted in the present study. The MOGQ assesses seven distinct gaming motivational factors through 27 items answered on a five-point Likert scale ranging from 1 (‘almost never/never’) to 5 (‘almost always/always’). The seven motivational factors are assessed via four items each and they include social, escape, competition, coping, skill development, fantasy and recreation gaming motives. Total scores for the MOGQ can be obtained by summing the participants’ answers to all four items of each motive (range of each subscale = 4 to 20 points; except recreation which ranges from 3 to 15 points), with higher scores indicating higher tendencies to each respective gaming motive.

##### Psychopathological Symptoms

In line with previous similar research [5] conducted in Germany, the study utilized previously validated psychometric tests to assess a wide range of psychopathological symptoms, including depression (Patient Health Questionnaire-9 (PHQ-9) [44]), loneliness (UCLA Loneliness Scale, version 3 [45]) and attention problems (Attention Problems Scale [46]). For the PHQ-9, the suicide item was excluded (due to ethical constraints in the sample recruited) and the remaining eight items were administered with a four-point Likert scale ranging from 0 (‘never’) to 3 (‘nearly every day’). The UCLA Loneliness Scale, version 3 includes three items rated on a four-point Likert scale ranging from 1 (‘never’) to 4 (‘often’). Moreover, the Attention Problems Scale includes three items answered on a five-point Likert scale ranging from 1 (‘strongly disagree’) to 5 (‘strongly agree’). Total scores across all psychopathological symptoms’ scales were obtained through summation of participants’ answers to all items of each scale, with higher scores indicating higher problem-severity on the specific symptom measured. In the present study, all three scales assessing psychopathological symptoms exhibited adequate levels of reliability (PHQ9-9: α = 0.80; UCLA Loneliness Scale, version 3: α = 0.86; and Attention Problems Scale: α = 0.73).

### 2.3. Statistical Analyses

The analysis strategy included a twofold data-analytic approach. More specifically, (i) descriptive statistics of the sample and in-depth comparative analysis of the prevalence rates of GD across both diagnostic frameworks and (ii) a complex mediation analysis to investigate the mediational role of gaming motives in the relationship between psychopathological symptoms and GD across the two diagnostic frameworks.

To assess the quality of the structural models, conventional Goodness of Fit (GOF) indices and accepted thresholds were adopted. These included the Comparative Fit Index (CFI); the Tucker-Lewis Fit Index (TLI) (90, 95); and the Root Mean Square Error of Approximation (RMSEA) (90% CI 0.05; 0.08) [47]. The CFA models were estimated with Robust Maximum Likelihood Estimator (MLR), to account for the multivariate distribution of the sample whereas the mediational models were estimated with the Maximum Likelihood Estimator (ML) using 5000 bootstrap samples to yield robust standardized errors [48]. All statistical analyses were performed using IBM SPSS Statistics Version 25 (IBM Corporation, New York, NY, USA) and M*plus* Version 8.3. (Statmodel, Los Angeles, CA, USA).

## 3. Results

### 3.1. Descriptive Statistics and Comparative Prevalence Rates Analyses

In terms of gender distribution, males represented 80% (*n* = 1229) of all participants. The mean age observed in the sample was 29.74 years (SD = 12.37 years, range = 12–82 years). Furthermore, about 52.41% (*n* = 749) of sample reported not being in a romantic relationship. With regards to employment status among the participants, 68.99% (*n* = 986) reported being unemployed at the time of the survey.

In terms of gaming-related behaviors, the average time spent gaming during the week was 19.15 h (SD = 14.69 h), with about 42% of this time being spent over the weekend alone. In terms of preferred mode of play, 55.84% (*n* = 798) of all participants reported playing mainly online games. Moreover, about 38.34% (*n* = 548) of the sample reported in-game social membership to a group (e.g., clan, guild). Although no participant self-identified as a professional gamer, a small minority (i.e., 5.74%, *n* = 82) demonstrated intentions to become a professional gamer in the future. Finally, about 10.5% (*n* = 150) of the respondents declared having experienced significant problems in their lives due to gaming.

Participants’ levels of psychopathological symptoms were also assessed in the sample, these included attention problems (mean = 6.43; SD = 2.52; min = 3, max = 15), depression (mean = 13.58; SD = 4.13; min = 8, max = 32), loneliness (mean = 6.12; SD = 2.98; min = 3, max = 15) and severity of GD across both diagnostic frameworks (APA mean = 19.36; SD = 6.62; min = 9, max = 44; WHO mean = 8.46; SD = 3.42; min = 4, max = 20). A complete summary of the sample’s main sociodemographic characteristics and severity of psychopathological symptoms is presented in Table 1.

The comparative analyses of the sample’s prevalence rates of GD according to each diagnostic framework revealed that about 5.74% (*n* = 82) of all participants fulfilled the APA criteria for IGD and about 3.28% (*n* = 47) met the WHO diagnostic criteria for GD. Interestingly, further analysis suggested that these two prevalence rates were significantly different as per the choice of the diagnostic framework (χ^2^ = 798.32, df = 1, *p* ≤ 0.001; φ = 0.75). Moreover, the ratio of gamers afflicted with GD did not differ across genders in relation to the APA (χ^2^ = 2.15, df = 1, *p* = 0.14) and the WHO frameworks (χ^2^ = 2.34, df = 1, *p* = 0.13). Age-related effects were found in the APA (*t*_(1427)_ = 3.84, *p* < 0.001) but not in the WHO framework (*t*_(1427)_ = 0.58, *p* = 0.56).

### 3.2. Examining the Mediational Role of Gaming Motives in the Relationship between Psychopathological Symptoms and GD

In order to further investigate the potential psychometric differences in the APA and WHO frameworks, a complex mediation analysis was conducted to compare how in-game motives may mediate the relationship between psychopathological symptoms and GD. All latent variables were included in two single structural equation models in which GD was predicted by depression, loneliness and attention problems through specific gaming motives namely social, escape, competition, coping, skill development, fantasy and recreation motives. To ensure the robustness of the findings, the computation of the two mediation models accounted for potential confounding effects stemming from time spent playing video games during the week (β_GDT_ = 0.25, *p* < 0.001; β_IGDS9-SF_ = 0.22, *p* < 0.001), age (β_GDT_ = −0.08, *p* < 0.001; β_IGDS9-SF_ = −0.09, *p* < 0.001) and gender (β_GDTref:female_ = −0.06, *p* = 0.01; β_IGDS9-SFref:female_ =−1.55, *p* = 0.11). Furthermore, the two mediation models were estimated using the ML estimator [49] with 5000 bootstrap samples to enhance the quality of the findings. The results of this analysis indicated that both mediation models presented an adequate fit to the data as evidenced by the following fit indices: ML_(GDT)_χ^2^_(__986)_ = 5488.32, CFI = 0.91, TLI = 0.90 and RMSEA = 0.05 (90% CI (0.05–0.05)); and ML_(IGDS9-SF)_χ^2^_(1226)_ = 6941.63, CFI = 0.90, TLI = 0.90 and RMSEA = 0.05 (90% CI (0.05–0.06)).

A more fine-grained analysis of the mediation results obtained (see Table 2 for a complete summary) suggested that across both diagnostic frameworks, depression had significant direct effects on the following gaming motives: social, escape, competition, coping, skill development, fantasy and recreation (β ranging from 0.30 to 0.81, *p* < 0.001). Additionally, loneliness (β ranging from 0.08 to 0.52, *p* ≤ 0.01) and attention problems (β ranging from 0.51 to 0.78, *p* < 0.001) also yielded statistically significant direct effects on all seven gaming motives. Overall, the results pertaining to the direct effects in the two mediation models were highly comparable across both diagnostic frameworks.

In relation to the mediational role of gaming motives on GD, the escape motive positively influenced GD with results closely matching across both diagnostic frameworks (β_GDT_ = 0.44, β_IGDS9-SF_ = 0.47, *p* < 0.001). Although the competition motive had a statistically significant effect on GD (β_GDT_ = 0.16, β_IGDS9-SF_ = 0.21, *p* < 0.001), this effect was rather weak. Moreover, negative direct effects on GD were found for both skill development (β_GDT_ = −0.09, β_IGDS9-SF_ = −0.08, *p* ≤ 0.006) and recreation motives (β_GDT_ = −0.14, β_IGDS9-SF_ = −0.09, *p* ≤ 0.004) across both diagnostic frameworks. Non-significant direct effects included the effects from social and fantasy motives on GD (*p* ≥ 0.16) across both diagnostic frameworks. Interestingly, the main difference observed across the two diagnostic frameworks was in relation to the predictive role of coping on GD as it had a weak influence on GD according to the APA (β = 0.08, *p* = 0.03) but not to the WHO framework (*p =* 0.08).

Overall, the results related to the indirect effects on the association between depression, loneliness and attention problems on GD through gaming motives yielded highly comparable results across the two diagnostic frameworks. More specifically, positive indirect effects of depression on GD through gaming motives were statistically significant for both escape and competition motives (ind. β ranging from 0.04 to 0.39, *p* < 0.001), whereas negative indirect effects were found through skill development and recreation motives (ind. β ranging from −0.02 to −0.04, *p* ≤ 0.01). Conversely, non-significant indirect paths were found across both diagnostic frameworks for social and fantasy motives as mediators (*p* ≥ 0.16). The only pronounced discrepancies found between the two diagnostic frameworks in terms of indirect effects occurred in the relationship between depression on GD through coping motive as it had a weak influence on GD according to the APA (ind.β = 0.05, *p* = 0.03) but not to the WHO framework (*p =* 0.08).

Positive indirect effects of loneliness on GD through gaming motives were statistically significant for escape, competition and coping motives only (ind.β ranging from 0.02 to 0.23, *p* ≤ 0.01). Additionally, weak statistically significant negative effects were found for skill development and recreation motives (ind.β = −0.01, *p* ≤ 0.04), while social motive had no mediational effect on the relationship between loneliness and GD (*p* ≥ 15). The indirect effects based on loneliness as a predictor also yielded highly consistent findings across both diagnostic frameworks. Furthermore, the only main discrepancy on the indirect effect of loneliness on GD emerged in relation to fantasy motive which produced a weak influence on GD according to the APA (ind.β = 0.02, *p* ≤ 0.04) but not to the WHO framework (*p =* 0.57).

As per the indirect effects in the relationship between attention problems and GD through all seven gaming motives, the results indicated positive and statistically significant indirect effects for escape, competition and coping motives (ind.β ranging from 0.08 to 0.30, *p* ≤ 0.02). Furthermore, negative indirect effects were found for skill development and recreation motives (ind.β ranging from −0.07 to −0.11, *p* ≤ 0.01). Interestingly, only two mediators related to social and fantasy motives had no mediational effects on the relationship between attention problems and GD (*p* ≥ 0.12). In short, the examination of the indirect effects in the two models indicated similar and highly consistent findings across both diagnostic frameworks.

Finally, the analysis of the total effects across both diagnostic frameworks identified slightly stronger effects for the APA framework in comparison to the WHO framework with depression (β_GDT_ = 0.39, *p* < 0.001; β_IGDS9-SF_ = 0.49, *p* < 0.001), loneliness (β_GDT_ = 0.24, *p* < 0.001; β_IGDS9-SF_ = 0.31, *p* < 0.001) and attention problems (β_GDT_ = 0.28, *p* < 0.001; β_IGDS9-SF_ = 0.41, *p* < 0.001) all positively influencing GD. Despite those minor differences at the level of total effects, these findings are highly comparable across both diagnostic frameworks.

## 4. Discussion

The present study used a large sample of German gamers to explore whether potential psychometric discrepancies exist between the APA and the WHO diagnostic framework for disordered gaming at different levels. Investigating such a research question is paramount for several reasons. Firstly, it is unclear to what extent the obvious diagnostic differences pertaining to both the APA and WHO frameworks may have an effect on the way in which GD intertwines with psychopathological symptoms, gaming motives and how such discrepancies may lead to potential bias in the estimation of prevalence rates of disordered gaming in future research. Secondly, the present study may help inform the development of new health policies on GD related to the refinement of diagnostic practices in disordered gaming by providing empirical data on the performance of both diagnostic frameworks. Thirdly, although not directly related to the main aims of the present study, the additional analyses on the psychometric properties of the German GDT and IGDS9-SF as reported in the Supplementary File also add to the cross-cultural knowledge base on GD by promoting a much-needed unified psychometric assessment [50,51] framework for GD by developing further two widely used psychometric tests for GD.

The present study also provides key empirical insights on the potential feasibility of the APA and WHO diagnostic frameworks for disordered gaming. This is particularly important given the lack of such studies and the generalized scholarly disagreement on this topic. A recent study by Ferguson and Colwell [52] found that among 214 scholars actively engaged in GD research, 60.8% agreed that GD could be a mental health problem. However, only 49.7% of the scholars sampled believed the APA framework and its nine diagnostic criteria were valid, while 56.5% demonstrated support to the WHO framework for GD [52]. The obvious lack of consensus among scholars warrants further empirically driven analysis of the APA and WHO diagnostic frameworks given that little is known about their comparative psychometric feasibility.

When comparing the performance of the APA and the WHO frameworks at the prevalence level, the findings obtained suggested that the WHO framework produced slightly more conservative prevalence rates for GD (i.e., 3.28%) in comparison to the APA framework (i.e., 5.74%). Although these prevalence rates are well aligned with the larger body of international epidemiological research using robust methodologies to assess disordered gaming (see Pontes [10] for a detailed review), the most important aspect of this finding was that these minor differences were statistically significant (as per the findings obtained in this study). Therefore, this has important implications for large-scale epidemiological research on GD as the choice of the psychometric tool to assess GD will likely inflate or deflate prevalence rates on the basis of the underlying diagnostic framework being operationalized in the existing psychometric tools to assess disordered gaming. It is worth noting that the observed effects on the prevalence rates of GD as per the diagnostic framework chosen does not imply that previous epidemiological research on IGD is scientifically flawed or invalid. Instead, the findings reported in this study suggest that future epidemiological research on GD should be mindful of potential discrepancies in their estimations of prevalence rates given the choice of the diagnostic framework utilized. The authors’ of the present study agree with the view that active efforts from multiple stakeholders (e.g., researchers, clinicians and health professionals) should be in place to avoid over pathologizing everyday life behaviors such as gaming [53]. Consequently, the findings obtained support the recommendation that more stringent and conservative assessment approaches capable of producing more realistic prevalence rates ought to be adopted in future research to mitigate the propensity for inflation (or deflation) of the actual prevalence rates across different populations of gamers worldwide. On a related note, the prevalence rates of GD reported in this study do not stem from a representative (gaming) sample, therefore self-selection bias in the recruitment process due to survey advertisement in the mass media might have had an influence on the estimates reported.

Recent recommendations by psychometricians actively engaged in GD research suggest [51] that adequate scrutiny of the psychometric properties of scales utilized to assess GD are still required in order to enhance the accuracy in the estimation of problem-severity, prevalence and incidence rates of GD as this will likely improve cross-country comparability in international research conducted in multiple countries.

An additional goal of the present study was to compare the psychometric performance of the APA and the WHO diagnostic frameworks through complex mediation analyses. The mediation models tested were all informed by previous research suggesting that gaming motives are linked to GD and that they mediate the relationship between psychopathological symptoms and GD [14,24,31,54,55,56]. Overall, the findings corroborated the existing evidence suggesting that gaming motives play an important role in mediating the relationship between psychopathological symptoms (such as depression [57], loneliness [58] and attention problems [59]) and GD even when analyzed according to different diagnostic frameworks for GD. Taken together, the mediation analyses indicated that gaming motives play an important role in mediating the relationship between several psychopathological symptoms and GD regardless of the diagnostic framework employed to diagnose GD.

Additionally, the mediation findings suggested that the indirect effects from the gaming motives on the relationship between different psychopathological symptoms and GD were highly consistent across the APA and WHO diagnostic frameworks. Nevertheless, three minor discrepancies due to the diagnostic framework emerged in the mediation models related to the direct effects of coping motive on GD and to the indirect effects of coping motive in the relationship between depression and GD and the fantasy motive in the relationship between loneliness and GD. Although these findings may suggest that the WHO framework is relatively stricter in detecting such effects in comparison to the APA framework, caution is advised when inferring such conclusions as the present study does not allow the inference of robust conclusions regarding whether the discrepancies observed at the mediational level exist due to actual real differences in the diagnostic frameworks or due to potential biases stemming from the analytical methods employed as significance levels (*p*-values), which are generally influenced by sample size as larger samples, tend to produce greater likelihood of positive findings (i.e., *p* < 0.05) [60].

In terms of direct effects stemming from gaming motives on GD, the present study found that specific gaming motives can constitute risk factors for GD as the escape motive was the strongest predictor of GD, with the competition motive presenting the second strongest predictive effect. These findings corroborate and expand upon previous similar research focusing on only one diagnostic framework for GD showing that the escape motive is the strongest predictor of GD [54,55,56,61,62] and that by contrast, competition produces a slightly weaker and yet significant influence on GD symptoms [27,55,56]. Interestingly, the present findings also illustrate that certain gaming motives may act as protective factors due to their negative predictive role of GD. More specifically, recreation and skill development motives were inversely linked to GD symptoms, a finding that is consistent with the extant evidence suggesting that gamers endorsing high levels of recreation [24] and skill development [55,56,62] exhibit lower levels of GD symptomatology in comparison to other gaming motives [27].

At the theoretical and clinical levels, the findings concerning the role of the escape motive in the development of GD can be framed within the self-medication hypothesis, which is used as a theoretical model to better understand the emergence of addictive disorders in the context of comorbidity [63,64,65]. The self-medication hypothesis posits that individuals engaging in addictive behaviors in relation to substance abuse can achieve relief for their ongoing emotionally painful states through such excessive behaviors. Furthermore, self-medication factors can occur in the context of deficient self-regulation patterns including but not limited to difficulties in regulating affects, self-esteem, social relationships and self-care [63,64,65]. Given that GD has been identified in previous empirical research as a potential stress response [66] that may be used as a coping process for real life problems [67], the self-medication hypothesis can thus be validly applied to the context of GD to generate a better understanding about this condition. The application of the self-medication hypothesis to GD receives support from clinical case studies [68] showing the validity of the self-medication hypothesis in GD as disordered gamers may engage in harmful gaming behaviors in an attempt to overcome psychological distress, leading up to excessive amounts of time spent playing and the emergence of functional impairment and additional comorbidities that aggravate the behavioral patterns toward gaming.

Even though the mediation findings were consistent and paralleled those of previous studies, caution is advised when interpreting the results reported in this study in relation to the specific etiological role of gaming motives in the development of GD symptoms. Limitations may exist due to the cross-sectional design of the study which does not allow such causal inferences. Additionally, gaming motives must also be investigated within specific contexts of gameplay. For example, it is unclear whether specific motivational patterns found in previous research using normative samples of gamers can be validly extended to specific gaming contexts such as professional gaming. Therefore, further research should be conducted to examine the mediational role of gaming motives in the relationship between mental health and GD within specific gaming populations such as professional gamers or highly competitive gamers in the context of e-sports. Further potential limitations in the present findings may be related to the sampling technique adopted (i.e., convenience sample of German gamers as opposed to a random sample), which can affect the extent to which the findings obtained can be generalized to the broader German population or even other cultural contexts. This is particularly relevant in the present study given the sampling technique utilized and how prevalence rates were produced on that basis. Additionally, the results may have been affected by potential biases stemming from the use of self-report measures, such as memory recall bias and social desirability. To overcome these potential issues, future research should attempt to collect data from diverse gaming communities (e.g., professional gamers) and employ more sophisticate designs (e.g., longitudinal design) to ascertain the temporal stability of the findings presented in this study. Future research should also aim to clarify the role of gaming motives in predicting GD across specific clinical groups as the endorsement of specific gaming motives can vary according to pre-existing comorbidities such as Autism Spectrum Disorder [69]. Finally, although the escape motive has been long established as a key predictor and risk factor for GD on its own, it is unclear to what extent the different facets of escape motives may produce differential effects in predicting GD as the studies conducted to date generally do not differentiate between the various facets of escapism. Therefore, the present study paves the way to future research aiming to investigate whether positive and negative escapism [70] may produce distinct effects on the development and severity of GD.

## 5. Conclusions

Despite the potential limitations considered above, the findings and general aims of the present study align with the international recommendation that additional research on GD is still needed to identify its key defining features, obtain cross-cultural data on the validity and reliability of existing psychometric tools, determine prevalence rates in countries around the world, evaluate the natural history and associated biological features of GD [71]. Moreover, the present study supports the conceptual and psychometric feasibility of the new WHO framework to assess disordered gaming behaviors given that the findings obtained in the present study in relation to the WHO framework were highly consistent with the APA framework that has been established earlier. In summary, the differences observed in the mediation analyses were rather marginal and may reflect limitations within the statistical approach utilized rather than real differences pertaining to the choice of the diagnostic framework. Moreover, the mediation analyses conducted contribute to expanding the international literature due to the focus given toward comparing simultaneously both diagnostic frameworks for GD.

In terms of potential clinical implications capable of informing emerging prevention and treatment policies, the present study substantiates the notion that gaming motives play an important role in the clinical assessment of GD and that assessing underlying gaming motives should be an inherent aspect of the assessment of GD behaviors due to its implication in the etiology, clinical course and treatment of GD as has been previously suggested [51,72]. In line with previous studies, the findings obtained suggest that in the context of treatment, it is essential that the clinician focuses on the motives driving their clients to engage in disordered gaming behaviors as opposed to focusing on the time they spend gaming as time spent gaming can be better used as a tool to evaluate the clients’ progress in the therapy and as an indication of the self-control exercised by the clients over disordered gaming [73]. With this in mind, it is paramount for future studies to investigate the intricacies between time spent playing and symptom-formation in the development of GD as there is no robust data to inform clinicians on where the draw the line between disordered and non-disordered gaming. Similarly, in the context of treatment, it is important that the clinician assists their clients in gaining a clear understanding of their life goals and gaming motives as this will prevent them from giving in to urges and cravings that are contrary to the goals of treatment, further providing their clients with a more complete and internally consistent sense of self [73].

## Figures and Tables

**Table 1:** Main sociodemographic characteristics and related gaming behaviors among the sample.

*N*	1429
Gender (Male, %)	1229 (80)
Age, Years (Mean, SD)	29.74 (12.37)
Relationship Status (in a Relationship, %)	749 (52.41)
Employment Status (Unemployed, %)	986 (68.99)
Time Spent Gaming on Weekdays (Mean, SD)	19.15 (14.69)
Time Spent Gaming on Weekends (%)	42
Preferred Mode of Play (online, %)	798 (55.84)
In-game Social Membership (Participation, %)	548 (38.34)
Intention to Become a Professional Gamer (yes, %)	82 (5.74)
Experienced Significant Problems due to Gaming (yes, %)	150 (10.5)
Attention Problems (Mean, SD)	6.43 (2.52)
Depression Severity (Mean, SD)	13.58 (4.13)
Loneliness Severity (Mean, SD)	6.12 (2.98)
GD Symptom Severity (Mean, SD)	8.46 (3.42)
IGD Symptom Severity (Mean, SD)	19.36 (6.62)
GD Prevalence (n, %)	47 (3.28)
IGD Prevalence (n, %)	82 (5.74)
Item-Related Descriptive Statistics (Mean, SD)	
GDT_item1_	2.29 (1.04)
GDT_item2_	2.60 (1.09)
GDT_item3_	1.95 (1.09)
GDT_item4_	1.62 (.92)
IGDS-SF9_item1_	2.53 (1.15)
IGDS-SF9_item2_	1.71 (1.01)
IGDS-SF9_item3_	1.94 (1.07)
IGDS-SF9 _item4_	1.90 (1.06)
IGDS-SF9 _item5_	1.89 (1.11)
IGDS-SF9_item6_	1.83 (1.09)
IGDS-SF9 _item7_	1.61 (.96)
IGDS-SF9_item8_	2.21 (1.21)
IGDS-SF9_item9_	1.34 (.80)

**Table 2:** Summary of the mediation analysis according to the World Health Organization (WHO) framework for Gaming Disorder (GD) and American Psychiatric Association (APA) framework for Internet Gaming Disorder (IGD).

	GD			IGD		
Direct Effects	β	*t*-value	*p*-value	β	*t*-value	*p*-value
Depression → Social	0.37	11.96	0.001	0.37	12.03	0.001
Depression → Escape	0.81	48.88	0.001	0.81	49.40	0.001
Depression → Competition	0.28	9.78	0.001	0.28	9.84	0.001
Depression → Coping	0.61	24.29	0.001	0.61	24.43	0.001
Depression → Skill development	0.34	11.67	0.001	0.34	11.72	0.001
Depression → Fantasy	0.62	26.33	0.001	0.62	26.57	0.001
Depression → Recreation	0.30	9.67	0.001	0.30	9.75	0.001
Loneliness → Social	0.17	5.43	0.001	0.17	5.50	0.001
Loneliness → Escape	0.52	21.85	0.001	0.52	22.13	0.001
Loneliness → Competition	0.12	4.26	0.001	0.13	4.36	0.001
Loneliness → Coping	0.33	11.36	0.001	0.33	11.44	0.001
Loneliness → Skill development	0.14	4.99	0.001	0.15	5.02	0.001
Loneliness → Fantasy	0.42	15.93	0.001	0.42	16.05	0.001
Loneliness → Recreation	0.08	2.59	0.01	0.08	2.66	0.01
Attention → Social	0.66	27.37	0.001	0.66	27.30	0.001
Attention → Escape	0.67	28.86	0.001	0.67	29.03	0.001
Attention → Competition	0.51	20.26	0.001	0.51	20.23	0.001
Attention → Coping	0.78	38.85	0.001	0.78	38.89	0.001
Attention → Skill development	0.66	30.78	0.001	0.66	30.67	0.001
Attention → Fantasy	0.68	30.19	0.001	0.68	30.27	0.001
Attention → Recreation	0.65	28.42	0.001	0.65	28.35	0.001
Social → GD	0.03	1.02	0.30	0.04	1.40	0.16
Escape → GD	0.44	10.47	0.001	0.47	12.28	0.001
Competition → GD	0.16	5.397	0.001	0.21	7.42	0.001
Coping → GD	0.07	1.74	0.08	0.08	2.10	0.03
Skill development → GD	−0.09	−2.78	0.005	−0.08	−2.76	0.006
Fantasy → GD	0.01	0.035	0.97	0.05	1.39	0.16
Recreation → GD	−0.14	−4.28	0.001	−0.09	−2.90	0.004
INDIRECT EFFECTS						
Depression → Social → GD	0.01	1.02	0.30	0.01	1.38	0.16
Depression → Escape → GD	0.36	9.86	0.001	0.39	11.43	0.001
Depression → Competition → GD	0.04	4.71	0.001	0.06	5.93	0.001
Depression → Coping → GD	0.04	1.73	0.08	0.05	2.08	0.03
Depression → Skill development → GD	−0.03	−2.69	0.007	−0.03	−2.67	0.01
Depression → Fantasy → GD	0.01	0.035	0.97	0.03	1.39	0.16
Depression → Recreation → GD	−0.04	−3.88	0.001	−0.02	−2.77	0.006
Loneliness → Social → GD	0.01	1.02	0.30	0.01	1.42	0.15
Loneliness → Escape → GD	0.21	9.35	0.001	0.23	10.74	0.001
Loneliness → Competition → GD	0.02	3.37	0.001	0.02	3.77	0.001
Loneliness → Coping → GD	0.02	2.08	0.03	0.03	2.57	0.01
Loneliness → Skill development → GD	−0.01	−2.36	0.01	−0.01	−2.36	0.01
Loneliness → Fantasy → GD	0.01	0.56	0.57	0.02	1.96	0.05
Loneliness → Recreation → GD	−0.01	−2.24	0.02	−0.01	−2.00	0.04
Attention problems → Social → GD	0.01	0.64	0.52	0.02	0.82	0.41
Attention problems → Escape → GD	0.28	9.32	0.001	0.30	10.80	0.001
Attention problems → Competition → GD	0.09	5.16	0.001	0.11	6.80	0.001
Attention problems → Coping → GD	0.07	2.02	0.01	0.08	2.28	0.02
Attention problems → Skill development → GD	−0.07	−2.84	0.01	−0.07	−2.98	0.003
Attention problems → Fantasy → GD	0.01	0.23	0.81	0.03	1.54	0.12
Attention problems → Recreation → GD	−0.11	−4.34	0.001	−0.07	−3.23	0.001
TOTAL EFFECTS						
Depression → GD	0.39	16.03	0.001	0.49	22.07	0.001
Loneliness → GD	0.24	12.81	0.001	0.31	15.92	0.001
Attention → GD	0.28	9.82	0.001	0.41	15.72	0.001
*Controls*						
Age → GD	−0.08	−3.33	0.001	−0.09	−3.90	0.001
Gender → GD	−0.06	−2.53	0.01	−0.03	−1.55	0.11
Gaming time → GD	0.25	9.43	0.001	0.22	8.94	0.001

Note: Additional correlation terms were added to the structural mediation models to achieve the reported fit indices.

## Supplementary Materials

The following are available online at https://www.mdpi.com/2077-0383/8/10/1691/s1.
