# Supplementary material for: Psychopathological Symptoms and Gaming Motives in Disordered Gaming—A Psychometric Comparison between the WHO and APA Diagnostic Frameworks
Source: J Clin Med. 2019 Oct 15;8(10):1691. doi: 10.3390/jcm8101691 (PMC6832511; doi:10.3390/jcm8101691)
Supplement: Supplementary file 1 [file jcm-08-01691-s001.pdf]

Psychopathological symptoms and gaming motives in disordered gaming: A psychometric comparison between the WHO and APA diagnostic frameworks

Rationale

This online supplementary information file provides the details regarding the psychometric validation of the German versions of the Gaming Disorder Test (GDT) [1] and the Internet Gaming Disorder Scale–Short-Form (IGDS9-SF) [2]. Given that one of the aims of study conducted was to make a contribution to the increasing cross-cultural knowledge base on the psychometric assessment of GD, analyses about the validity and reliability of the German GDT [1] and IGDS9-SF [2] were conducted to support its future use in German-based studies. These psychometric tools are among the most widely used in the assessment of GD across multiple countries (e.g., [3-7]).

The developed the German GDT and IGDS9-SF may help to promote unification in the assessment of GD and to build upon the rapidly expanding cross-cultural knowledge base on the psychometric assessment of GD aiming to utilize the world's first psychometric tool for GD based on the WHO diagnostic frameworks [1].

Psychometric validation statistical analysis

The psychometric properties examination of the German GDT and IGDS9-SF included assessment of validity using a Multiple Indicators Multiple Causes (MIMIC) model approach alongside a reliability analysis using different reliability indicators (i.e., Cronbach's alpha, Composite Reliability [CR], and Factor Determinacy [FD]).

Results of the psychometric validation of the German GDT and IGDS9-SF

The assessment of the scales' construct validity was carried out by examining the factor structure of both GD and IGD under a single-factor solution with four items for the German GDT and nine items of the German IGDS9-SF as previously reported in the international literature [1-4]. The validity of the scales was examined in two MIMIC models including both latent and observable variables (see Figure 1). In both models, GD and IGD were controlled for the potential confounding effects of age, gender, and weekly time spent playing video games. The choice of these control variables was informed by extensive empirical studies that reported GD to be associated with younger age, and male gender, and an increment in time spent playing video games [8-10].

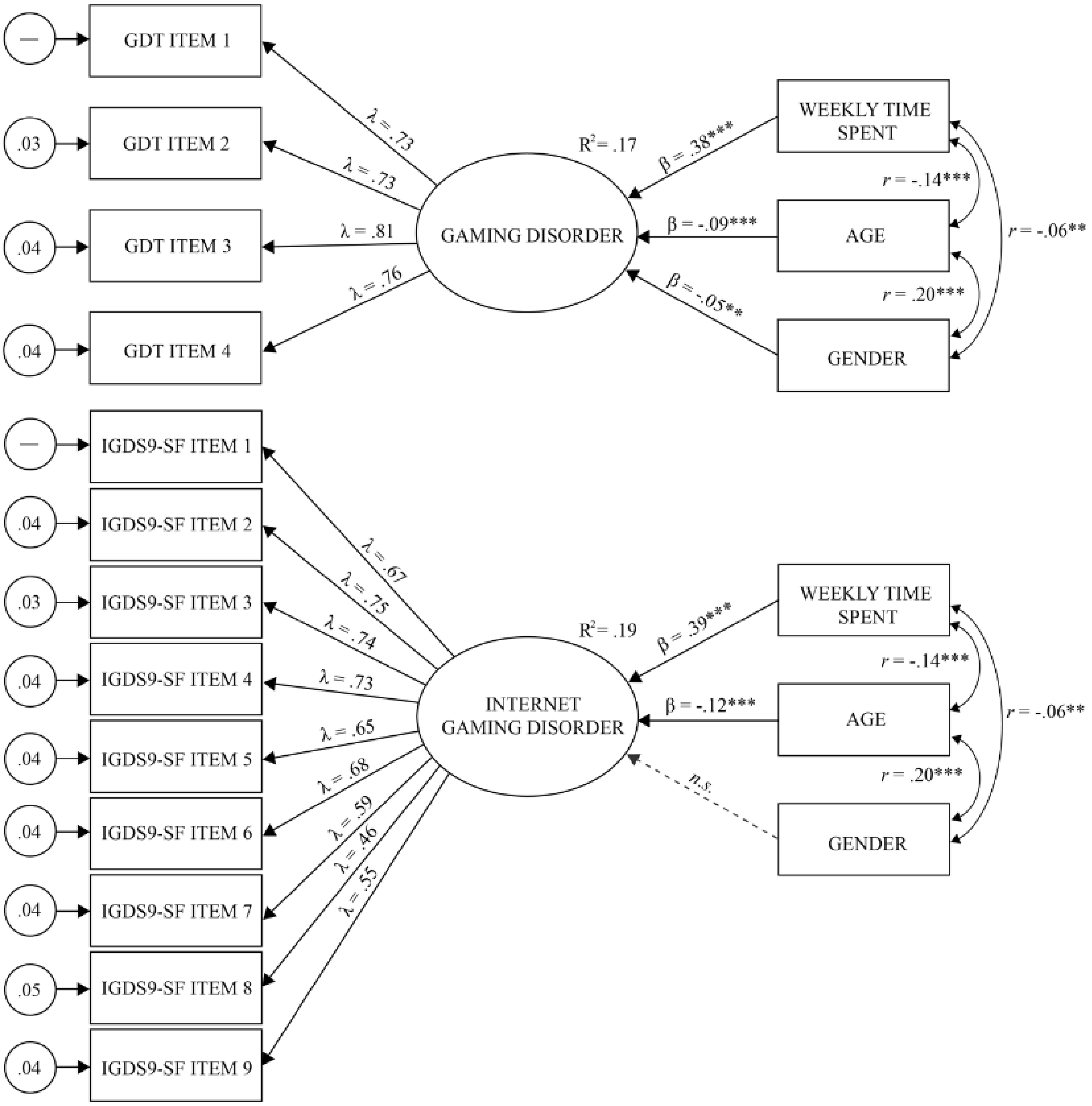

Figure 1. Gaming disorder – MIMIC models.

The results of the validity analysis yielded the following GOF for the German GDT: MLR$\chi^2_{(11)}$ = 105.16, CFI = .96, TLI = .92, and RMSEA = .07 (90% [CI .06–.08]); and for the German IGDS9-SF: MLR$\chi^2_{(51)}$ = 389.98, CFI = .91, TLI = .90, and RMSEA = 0.06 (90% CI [.06–.07]). These fit indices suggest that the unidimensional factor structure for GD and IGD as measured by both psychometric tests fit well the data. Moreover, the standardized item loadings for the GDT ranged from λ_{GDT2} = .73 to λ_{GDT3} = .81, whereas for the item loadings for the IGDS9-SF ranged from $\lambda_{IGDS9\text{-}SF8}$ = .46 to $\lambda_{IGDS9\text{-}SF3}$ = .75. All the standardized loadings were statistically significant and above the desired threshold of $\lambda_{ij} \geq .50$ [11], with the exception

of $\lambda_{IGDS9\text{-}SF8}$ (i.e., .46, $p < .05$). However, this was not deemed problematic as standardized item loadings between .45 and .54 are generally deemed ‘fair’ in social sciences [12].

Furthermore, the results indicated that GD was positively influenced by time spent playing video games during the week across both diagnostic frameworks ($\beta_{GDT} = .38$, $p < .001$; $\beta_{IGDS9\text{-}SF} = .38$, $p < .001$), as well as age ($\beta_{GDT} = -.09$, $p < .001$; $\beta_{IGDS9\text{-}SF} = -.12$, $p < .001$). The influence of gender on GD was statistically significant only for the WHO framework ($\beta_{GDTref:female} = -.05$, $p = .03$) but not for the APA framework ($p = .17$).

To further investigate the psychometric properties of the newly developed scales, several reliability indicators were computed to assess the reliability of the German versions of the GDT and IGDS9-SF. These included the Cronbach’s alpha, Composite Reliability (CR), and Factor Determinacy (FD). The Cronbach’s alphas of the GD were excellent for both the GDT ($\alpha = .84$) and the IGDS9-SF ($\alpha = .87$). Moreover, the CR coefficients for the GDT (CR = .84) and the IGDS9-SF (CR = .87) were equally satisfactory and well above the recommended threshold of .70 [11,13]. Finally, the estimation of the FD coefficients for the GDT (FD = .93) and IGDS9-SF (FD = .94) also produced reliability coefficients above the recommended threshold of .80 [14]. Taken together, these findings indicate that the German versions of the GDT and IGDS9-SF exhibit adequate levels of validity and reliability to measure GD symptoms based on both the APA and the WHO frameworks among German gamers.

References

1. Pontes, H.M.; Schivinski, B.; Sindermann, C.; Li, M.; Becker, B.; Zhou, M.; Montag, C. Measurement and conceptualization of Gaming Disorder according to the World Health Organization framework: The development of the Gaming Disorder Test. *International Journal of Mental Health and Addiction* **2019**, 10.1007/s11469-019-00088-z, doi:10.1007/s11469-019-00088-z.
2. Pontes, H.M.; Griffiths, M.D. Measuring DSM-5 Internet Gaming Disorder: Development and validation of a short psychometric scale. *Comput. Human Behav.* **2015**, *45*, 137-143, doi:10.1016/j.chb.2014.12.006.
3. Stavropoulos, V.; Beard, C.; Griffiths, M.D.; Buleigh, T.; Gomez, R.; Pontes, H.M. Measurement invariance of the Internet Gaming Disorder Scale–Short-Form (IGDS9-SF) between Australia, the USA, and the UK. *International Journal of Mental Health and Addiction* **2018**, *16*, 377-392, doi:10.1007/s11469-017-9786-3.
4. Pontes, H.M.; Stavropoulos, V.; Griffiths, M.D. Measurement Invariance of the Internet Gaming Disorder Scale–Short-Form (IGDS9-SF) between the United States of America, India and the United Kingdom. *Psychiatry Res.* **2017**, *257*, 472-478, doi:10.1016/j.psychres.2017.08.013.
5. Schivinski, B.; Brzozowska-Woś, M.; Buchanan, E.M.; Griffiths, M.D.; Pontes, H.M. Psychometric assessment of the Internet Gaming Disorder diagnostic criteria: An Item Response Theory study. *Addictive Behaviors Reports* **2018**, 10.1016/j.abrep.2018.06.004, doi:10.1016/j.abrep.2018.06.004.
6. Gomez, R.; Stavropoulos, V.; Beard, C.; Pontes, H.M. Item Response Theory analysis of the recoded Internet Gaming Disorder Scale-Short-Form (IGDS9-SF). *International Journal of Mental Health and Addiction* **2019**, *17*, 859-879, doi:10.1007/s11469-018-9890-z.
7. Pontes, H.M.; Griffiths, M.D. Portuguese validation of the Internet Gaming Disorder Scale–Short-Form. *Cyberpsychol Behav Soc Netw* **2016**, *19*, 288-293, doi:10.1089/cyber.2015.0605.
8. Fuster, H.; Carbonell, X.; Pontes, H.M.; Griffiths, M.D. Spanish validation of the Internet Gaming Disorder-20 (IGD-20) Test. *Comput. Human Behav.* **2016**, *56*, 215-224, doi:10.1016/j.chb.2015.11.050.
9. Lemmens, J.S.; Valkenburg, P.M.; Peter, J. Development and validation of a game addiction scale for adolescents. *Media Psychol.* **2009**, *12*, 77-95, doi:10.1080/15213260802669458.
10. de Palo, V.; Monacis, L.; Sinatra, M.; Griffiths, M.D.; Pontes, H.M.; Petro, M.; Miceli, S. Measurement invariance of the nine-item Internet Gaming Disorder Scale (IGDS9-SF) Across Albania, USA, UK, and Italy. *International Journal of Mental Health and Addiction* **2018**, 10.1007/s11469-018-9925-5, doi:10.1007/s11469-018-9925-5.
11. Hair, J.F.; Black, W.C.; Babin, B.J.; Anderson, R.E. *Multivariate data analysis. A global perspective (seventh edition)*; Pearson Prentice Hall: Upper Saddle River, CA, 2010.
12. Tabachnick, B.G.; Fidell, L.S. *Using multivariate statistics (sixth edition)*; Pearson Education: Essex, England, 2013.
13. Fornell, C.; Larcker, D.F. Evaluating structural equation models with unobservable variables and measurement error. *Journal of Marketing Research* **1981**, *18*, 39-50, doi:10.2307/3151312.
14. Muthén, L.K.; Muthén, B.O. *Mplus user's guide (seventh edition)*; Muthén & Muthén: Los Angeles, CA, 2012.
